# Reconfigurable Surface Micropatterns Based on the Magnetic Field-Induced Shape Memory Effect in Magnetoactive Elastomers

**DOI:** 10.3390/polym13244422

**Published:** 2021-12-16

**Authors:** Matija Lovšin, Dominik Brandl, Gašper Glavan, Inna A. Belyaeva, Luka Cmok, Lucija Čoga, Mitjan Kalin, Mikhail Shamonin, Irena Drevenšek-Olenik

**Affiliations:** 1 Department of Complex Matter, J. Stefan Institute, Jamova 39, 1000 Ljubljana, Slovenia; matija.lovsin@ijs.si (M.L.); luka.cmok@ijs.si (L.C.); 2 East Bavarian Centre for Intelligent Materials (EBACIM), Ostbayerische Technische Hochschule (OTH) Regensburg, Seybothstr. 2, 93053 Regensburg, Germany; dominik1.brandl@st.oth-regensburg.de (D.B.); gasper.glavan@oth-regensburg.de (G.G.); inna.belyaeva@oth-regensburg.de (I.A.B.); mikhail.chamonine@oth-regensburg.de (M.S.); 3 Faculty of Mechanical Engineering, University of Ljubljana, Aškerčeva 6, 1000 Ljubljana, Slovenia; Lucija.Coga@fs.uni-lj.si (L.Č.); mitjan.kalin@fs.uni-lj.si (M.K.); 4 Faculty of Mathematics and Physics, University of Ljubljana, Jadranska 19, 1000 Ljubljana, Slovenia

**Keywords:** magnetoactive elastomers, shape memory effect, surface microstructuring, optical diffraction

## Abstract

A surface relief grating with a period of 30 µm is embossed onto the surface of magnetoactive elastomer (MAE) samples in the presence of a moderate magnetic field of about 180 mT. The grating, which is represented as a set of parallel stripes with two different amplitude reflectivity coefficients, is detected via diffraction of a laser beam in the reflection configuration. Due to the magnetic-field-induced plasticity effect, the grating persists on the MAE surface for at least 90 h if the magnetic field remains present. When the magnetic field is removed, the diffraction efficiency vanishes in a few minutes. The described effect is much more pronounced in MAE samples with larger content of iron filler (80 wt%) than in the samples with lower content of iron filler (70 wt%). A simple theoretical model is proposed to describe the observed dependence of the diffraction efficiency on the applied magnetic field. Possible applications of MAEs as magnetically reconfigurable diffractive optical elements are discussed. It is proposed that the described experimental method can be used as a convenient tool for investigations of the dynamics of magnetically induced plasticity of MAEs on the micrometer scale.

## 1. Introduction

Surface properties play a vital role in many physical processes. They govern interfacial phenomena, such as friction, adhesion and wettability. They also determine the interactive properties of the objects with optical radiation, like objects’ color appearance and shine. One of the essential surface features is surface topography. Practically any mechanism that possesses a capacity to modify surface topography also affects the phenomena mentioned above.

Magnetoactive elastomers (MAEs) are rubbery substances composed of a compliant polymer matrix with embedded micrometer-sized ferromagnetic particles [1,2,3,4,5]. They are also known as magnetorheological elastomers (MREs) and are often perceived as solid analogs to magnetorheological fluids [6,7,8]. The exposure of MAEs to a magnetic field is an intriguing process that can induce profound changes in the surface topography of a compliant material [9,10]. In soft MAEs with Young’s modulus below 100 kPa (in the absence of a magnetic field) [11,12,13], a magnetic field applied in the direction perpendicular to the surface induces the formation of “mountain-like” structures [14], which resemble the Rosensweig peaks in ferrofluids and produce a strong increase in surface roughness [15,16,17,18,19,20,21,22,23,24]. In our recent characterization of this effect, we found that the root mean square (RMS) roughness was in the range of 1 μm/T [25]. Other phenomena on MAE surfaces that were also demonstrated to be regulable by a magnetic field are wettability [14,15,26,27,28], adhesive properties [29,30,31,32], friction [33,34,35,36,37,38], drop splashing [39] and surface optical properties [21]. For the latter, it was demonstrated that the total optical reflectivity and the type of reflection (specular or diffuse) could be controlled by the magnetic field.

Exposure of MAEs to magnetic fields also provokes the appearance of another interesting phenomenon, namely the magnetically induced plasticity effect, also known as the magnetic shape memory effect [5,40,41,42,43,44,45,46]. This effect provides a possibility to temporary “freeze” the shape of a MAE object by exposing it to the magnetic field [47]. It also enables to temporary create different patterns in the MAE surface by the embossing process [48,49]. The embossed pattern persists while the magnetic field is present and fades away when the field is removed. The recovery happens due to the regained elasticity of the material that flattens the surface back to its initial shape. Afterward, another pattern can be imposed. 

Up to now, investigations of the shape memory effect in MAEs have been limited to macroscopic forms and patterns (size of several mm). In this work, we report on the magnetic regulation of a micropattern embossed in the MAE surface. The pattern had a periodic structure with the periodicity of 30 μm, due to which it could have been conveniently detected via diffraction of a laser beam. Its stability in a constant magnetic field, erasure with decreasing magnetic field and reconfigurability after the erasure process were analyzed. In addition, being a convenient tool for probing the micropatterning features, the explored optical diffraction is also interesting due to its possible applications in devices such as magnetically reconfigurable diffractive optical elements (DOEs), magnetically tunable optical sensors and magneto-optical feedback units.

## 2. Materials and Methods

The disk-shaped MAE samples with a diameter of 20 mm and a thickness of 2 mm were synthesized following the previously described synthesis routes [12,50]. The compositions containing 70 and 80 wt% of soft-magnetic carbonyl iron powder (CIP, type SQ, BASF SE Carbonyl Iron Powder & Metal Systems, Ludwigshafen, Germany) and exhibiting different stiffness in zero magnetic field were investigated. The incorporated CIP particles had a mean diameter of 4.5 µm and were uniformly distributed in the polymer matrix. Stamps for embossing were fabricated from the standard photoresist material (SU-8) that was spin-coated onto a glass plate and then structured into a pattern of periodic slabs by a laser direct imaging system (ProtoLaser LDI, LPFK Laser & Electronics, Garbsen, Germany). The slabs had a quadratic cross-section of 15 × 15 µm^2^ and were separated laterally for 15 µm from each other. Hence, they formed a one-dimensional periodic structure with the periodicity *Λ* = 30 µm. The patterned area had a size of 20 × 4 mm^2^.

A selected MAE sample was placed on the horizontally oriented nonmagnetic platform. Magnetic field *B* was applied to the sample by placing a NdFeB-type permanent magnet in size of 20 × 20 × 5 mm^3^ underneath the sample platform. The orientation of the field in the central region of the sample was perpendicular to the sample surface. The magnet was mounted on a vertical translation stage and the magnitude of the magnetic field in the sample region was varied by changing the distance between the magnet and the sample. Before the experiments, the setup was calibrated by measurement of the field strength at the sample position as a function of translation distance with a Hall sensor. The field was varied in the range from *B* = 5 mT to *B* = 180 mT. During the embossing process, the sample was exposed to *B* = 180 mT. The stamp for embossing was fixed on another vertical translation stage and placed above the sample. Then, it was slowly moved towards the top surface of the sample and gently pressed into the sample. The translation was stopped about 15 μm after the stamp had touched the surface. In the last part of the process, the stamp was carefully lifted back to its initial position.

The observations revealed that only samples with 80 wt% of CIP could have retained the embossed pattern for several hours, while for samples with 70 wt% of CIP the pattern vanished in about 5 min after the embossing process, regardless of the applied magnetic field. Therefore, only samples with 80% of CIP were used for further experiments. These samples were named M1, M2 and M3 and had the following values of the shear storage modulus at *B* = 0: 7.1 kPa (M1), 16.3 kPa (M2) and 25.5 kPa (M3), respectively. 

**Figure 1:**
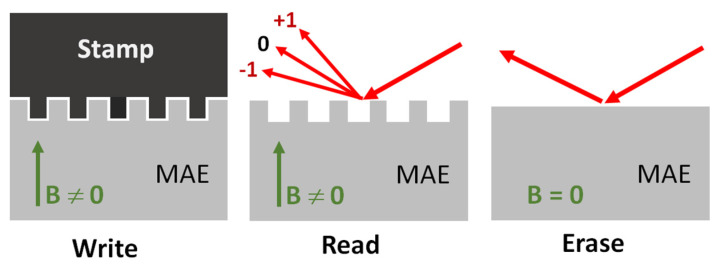
Schematic drawing of the writing (embossing), reading (optical diffraction) and erasure (transition to specular reflection) processes on the MAE surface.

Optical diffraction measurements were performed with a collimated beam from a He-Ne laser with the wavelength *λ* = 633 nm and output power of 5 mW (Uniphase, Manteca, CA, USA). The beam was linearly polarized in the direction parallel to the sample surface (TE polarization) and was impinging on the sample at the angle *θ* = 67° with respect to the surface normal. The transversal diameter of the beam on the surface was 1 mm. The far-field diffraction pattern was projected onto the opaque screen placed around 30 cm behind the sample. It was imaged by the monochrome CCD camera (Blackfly BFLY-U3-23S6M-C, FLIR, Wilsonville, OR, USA). The intensities *I*_0_, *I*_−1_ and *I*_+1_ of the specular reflected beam (0th order diffraction peak) and the −1st and the +1st order diffraction peaks were analyzed. The intensities of higher-order peaks were negligible. The ±1st order diffraction peaks were observed at the diffraction angles

(1) $$\gamma \approx \pm \frac{\lambda }{\Lambda cos\theta }=\pm 3.1{}^{\circ},$$

with respect to the direction of the specular reflection (see Figure 1). After background subtraction, the intensities of the three peaks were obtained by integration of the CCD signal within the three equal-sized areas corresponding to the area of the specular reflection. The diffraction efficiency was calculated as

(2) $$\eta_{m}=\frac{I_{m}}{I_{-1}+I_{0}+I_{+1}},\;m=-1,+1.$$

By this definition, which is frequently used in experiments performed on scattering media, the absorption and scattering losses of the probe beam are disregarded. We observed that the value of η_−1_ was sometimes somewhat larger than the value of η_+1_. So, to enable more relevant comparison between different experiments, we usually consider average diffraction efficiency <η> = (η_+1_ + η_−1_)/2.

Standard optical microscopy imaging (Nikon Optiphot2, Nikon, Tokyo, Japan) of the samples was performed with a long working distance objective with a 5× magnification. The magnet was kept under the sample all the time between the embossing and the imaging process. Optical profilometry measurements were performed with a 3D optical microscope (Bruker—ContourGT-K0, Billerica, MA, USA) using white-light interferometric objectives with 5× and 20× magnification. Cross-sectional profiles of the surface topography within the surface area of 400 × 400 μm^2^ were analyzed. The profilometer is based on scanning white-light interferometry, where the distance between the sample and the interferometric objective is automatically varied, while the corresponding micrographs showing the vertical displacement of interference fringes are recorded.

The basic idea for the performed investigations is schematically shown in Figure 1. The embossing process (“write” phase) takes place in the presence of a magnetic field and produces a surface micropattern of periodic channels that acts as an optical grating structure causing optical diffraction into the −1st and the +1st order diffraction peaks (“read” phase). This surface structure and the corresponding diffraction image persist so long as the magnetic field remains constant. When the magnetic field is removed, the diffraction vanishes and only specular reflection remains (“erase” phase).

## 3. Results

### 3.1. Experimental Results

**Figure 2:**
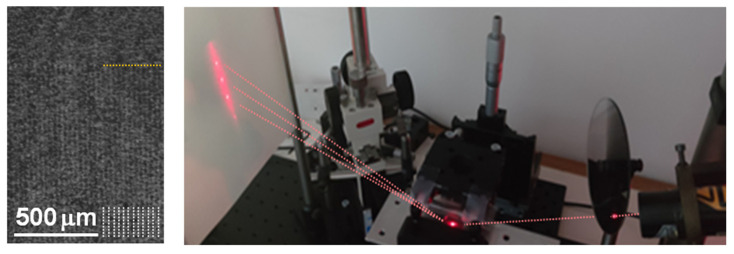
(**left**) Optical microscopy image of the micropatterned surface area. White vertical lines indicate grating lines. The border between the patterned and nonpatterned regions is indicated by a yellow horizontal line. (**right**) Photo of the experimental setup with a typical diffraction image projected onto a white screen. Red dotted lines indicate the paths of the incident and diffracted optical beams.

An optical microscopy image of the sample surface after the embossing process is shown on the left side of Figure 2. A periodic pattern in the form of vertical stripes can be noticed. A smaller upper part of the image corresponds to the nonpatterned region. The right side of Figure 2. shows the optical diffraction setup. The laser spot on the sample surface is seen as a red ellipse at the bottom of the figure. A far-field diffraction pattern with three diffraction peaks is observed on the observation screen. The peaks are surrounded by a “halo” of diffuse scattering.

**Figure 3:**
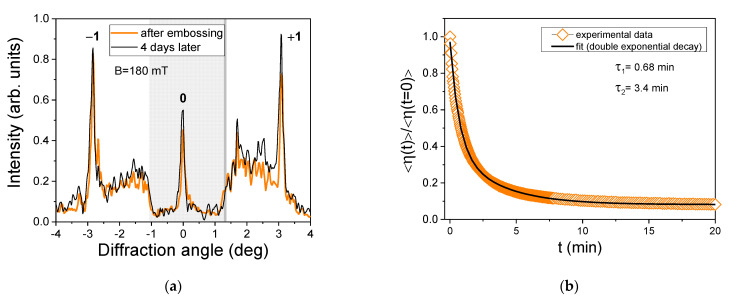
(**a**) Transversal profile of the far-field optical diffraction pattern recorded a few minutes (thick orange line) and four days (thin black line) after the embossing process in sample M3. To resolve more details, the optical signal in the central region (gray-shaded area) was attenuated for a factor of 5 with respect to other regions. The sample was constantly kept in the magnetic field *B* = 180 mT. (**b**) Time dependence of normalized diffraction efficiency <η(*t*)>/<η (*t* = 0)> after fast removal of the magnet. The solid line is a fit to a two-exponential decay function with decay times *τ*_1_ = 0.68 min and *τ*_2_ = 3.4 min.

The cross-section of the diffraction pattern for sample M3 is shown in Figure 3a. To increase the visibility of the ±1st order diffraction peaks, the intensity of the central (0th order) peak was attenuated for a factor of around 5. The thick orange line corresponds to the diffraction pattern recorded a few minutes after the embossing and the thin black line to the diffraction pattern recorded four days after the embossing. Between the two measurements, the sample was constantly exposed to the magnetic field of 180 mT. The diffraction features remained practically the same, indicating that the embossed microstructure was stable over more than 90 h. We also analyzed the decay of the diffraction efficiency after the magnet was quickly removed away from the sample. The result is shown in Figure 3b. The observed dependence was fitted to a two-exponential decay function. The obtained decay times are *τ*_1_ = 0.68 min and *τ*_2_ = 3.4 min.

**Figure 4:**
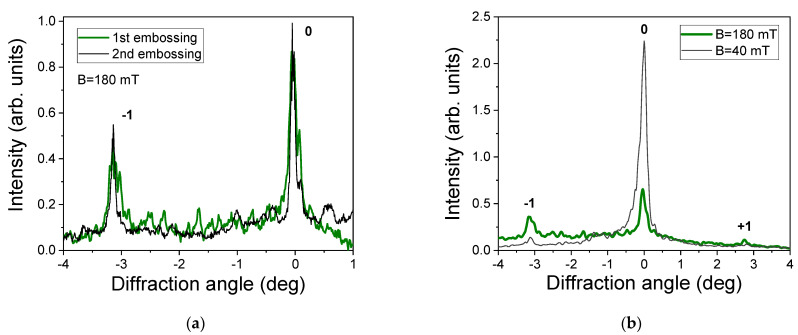
(**a**) Transversal profile of the far-field optical diffraction patterns observed as outcomes of two separate embossing processes in sample M2. Both diffraction measurements were performed at *B* = 180 T. In between the two embossing episodes, the sample was removed from the magnetic field for several hours. (**b**) Far-field optical diffraction pattern observed at *B* = 180 mT (thick green line) and at *B* = 40 mT (thin black line). The value of *B* was slowly reduced from 180 mT to 40 mT in steps of about 20 mT.

To examine the reconfigurability of the surface microstructure, we embossed and erased the pattern on the surface of the same sample several times. The orientation of the subsequent patterns was randomly varied. The observed optical diffraction patterns in two such experiments with sample M2 are shown in Figure 4a. The width of the diffraction peaks and the setting of diffuse scattering are slightly varied, while the ratio between the intensities of the −1st and the 0th order peaks is practically the same. This result demonstrates good reproducibility of the embossed surface microstructure.

In the next series of experiments, we investigated modifications of the diffraction efficiency during a step-by-step decrease of the magnetic field from *B* = 180 mT to *B* = 0. After every modification of the field, we let the structure stabilize for 10 min before the diffraction pattern was captured. Figure 4b shows the diffraction patterns obtained at *B* = 180 mT (green line) and *B* = 40 mT (black line). The intensity of the 0th order peak increases with the decreasing field, while the intensities of the ±1st order peaks decrease. Consequently, the diffraction efficiency decreases too. In addition to this, on these diffraction patterns also some asymmetry between the −1st and the +1st peak can be noticed.

**Figure 5:**
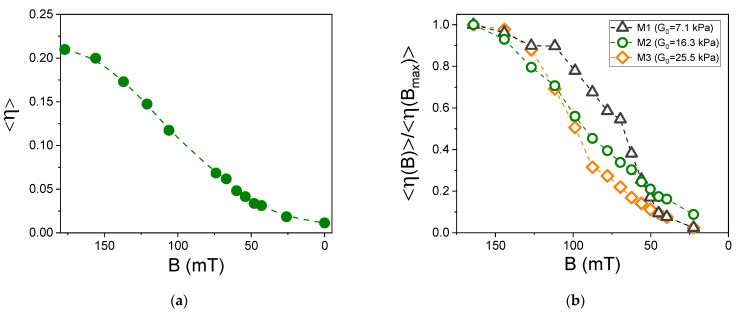
(**a**) Measured diffraction efficiency <η> as a function of magnetic field *B* for sample M2. (**b**) Normalized diffraction efficiency <η (*B*)>/<η (*B*_max_)> as a function of magnetic field *B* for three MAE samples with different matrix softness.

Figure 5a shows average diffraction efficiency <η> as a function of magnetic field *B* for sample M2. The value of <η> monotonically decreases with decreasing field and at *B* = 0 practically vanishes. Figure 5b gives a normalized diffraction efficiency <η(*B*)>/<η(*B*_max_)> as a function of *B* for all three investigated MAEs compositions. For the hardest sample (M3), a decrease of diffraction efficiency to 1/2 of its initial value occurs at *B*~100 mT, while for the softest sample (M1), it occurs at *B* ~ 60 mT. The medium-soft sample (M2) exhibits an intermediate behavior. This observation implies, as is expected, that the elasticity-driven erasure process is more efficient in hard than in soft samples. After the erasure process, if the magnetic field is increased again, the diffraction efficiency remains the same as at the end of the erasure process (these data are not shown).

**Figure 6:**
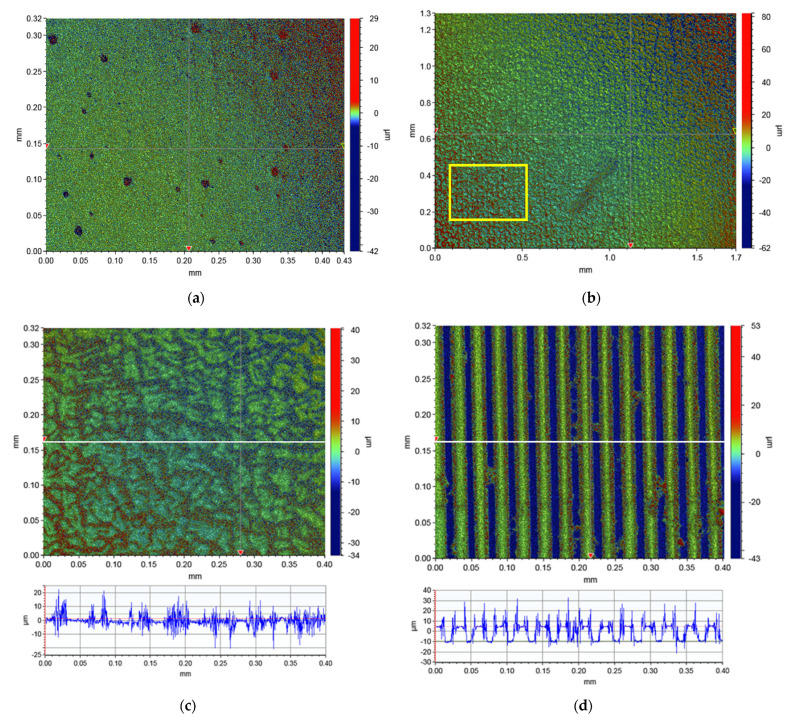
Optical profilometry images of (**a**) the surface of sample M1 before embossing in *B* = 0 and (**b**,**c**) after embossing in *B* = 180 mT. (**d**) Optical profilometry image of the stamp used for embossing. Graphs at the bottom of (**c**,**d**) are cross-sections along the white lines depicted in topographical images. Yellow square in (**b**) indicates the relative size of the surface areas shown in images (**a**,**c**,**d**).

The last series of experiments considered surface analysis with 3D optical profilometry. Figure 6a. shows the surface topography of sample M1 imaged in the absence of a magnetic field. The surface is very flat, only some “craters” due to air bubbles can be noticed. Figure 6b,c. show the surface of the same sample after the embossing process at two different magnifications. During the embossing and the subsequent imaging, the sample was constantly exposed to *B* = 180 mT. A periodic modulation of the surface topography is revealed. At the bottom of Figure 6c, a cross-sectional profile of surface features along the white horizontal line depicted in the topographical image is shown. The regions with a relatively flat surface landscape periodically exchange with the regions of very distorted surface structure. The distortions are in the vertical range of ±10 μm. For comparison, Figure 6d shows a topographical image and the cross-sectional profile of the stamp used for embossing. Periodic slabs with a height of 15 μm can be clearly resolved. However, one can notice that there appear many “spikes” at the edge regions of the slabs. We assume that this is an artifact associated with the white-light interferometry that generally has difficulties resolving sharp edges.

### 3.2. Model of the Optical Diffraction Structure

**Figure 7:**
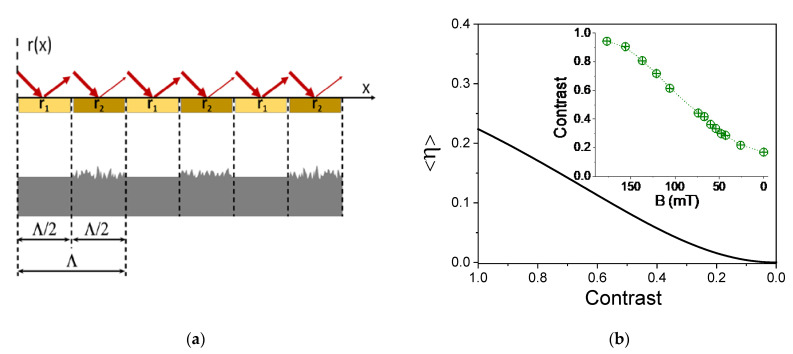
(**a**) Schematic drawing of the optical grating composed of parallel reflective stripes with amplitude reflectivity coefficients *r*_1_ and *r*_2_ < *r*_1_ (top drawing) and the corresponding MAE surface topography (bottom drawing). (**b**) Calculated dependence of diffraction efficiency *<*η*>* of the ±1st diffraction orders as a function of reflectivity contrast *c* = (*r*_1_ − *r*_2_)/(*r*_1_ + *r*_2_). The inset gives the contrast of the grating lines for sample M2 as a function of magnetic field *B* calculated on the basis of the experimental data shown in Figure 5a.

In accordance with the results of the 3D optical profilometry (Figure 6c), we propose to describe the imposed optical grating structure as a set of parallel stripes with two different amplitude reflectivity coefficients (*r*_1_ and *r*_2_). The schematic drawing of the model is shown in Figure 7a. The stripes with larger roughness are supposed to have a smaller value of *r*, so we assume *r*_2_ < *r*_1_. This is because larger roughness induces stronger spread optical reflection and consequently, less light is reflected in the direction of specular reflection, which effectively acts as a reduced reflectivity.

In the Fraunhofer diffraction theory, the diffraction pattern of such a structure is described as [51]:

(3) $$I(\gamma )\propto {\left(\frac{\sin\left(\frac{N\omega \Lambda }{2}\right)}{\sin\left(\frac{\omega \Lambda }{2}\right)}\right)}^{2}{\left|f(\gamma )\right|}^{2},$$

where the first term is the structure factor in which *ω* = 2*πγ*cos*θ*/*λ* is the spatial frequency and *N* is the number of illuminated grating periods, which in our setup was ~85 and *f*(*γ*) is the form factor. The latter is calculated as

(4) $$f\left(\gamma \right)=\overset{\Lambda }{\underset{0}{\int }}a\left(x\right)e^{i\omega x}dx\;,\;$$

where *a*(*x*) is the aperture function given as

(5) $$a\left(x\right)=\{\;\begin{array}{c} r_{1}\;;\;0<x<\Lambda /2\\ r_{2}\;;\;\Lambda /2<x<\Lambda \end{array}\;.$$

For such an aperture function, it follows:

(6) $${\left|f(\gamma )\right|}^{2}={\left(\frac{\Lambda }{2}\right)}^{2}{\left(\frac{\sin\left(\frac{\omega \Lambda }{4}\right)}{\frac{\omega \Lambda }{4}}\right)}^{2}\left[r_{1}^{2}+r_{2}^{2}+2r_{1}r_{2}\cos\left(\frac{\omega \Lambda }{2}\right)\right].$$

The diffraction maxima appear when the denominator in the first term of the Equation (3) is zero, i.e., when the condition *ω* = *m*(2*π*/*Λ*) is fulfilled, where *m* = 0, ±1, ±2, … denotes the diffraction order. The corresponding values of ${\left|f\left(\gamma \right)\right|}^{2}$, which determine the intensities of the peaks, are:

(7) $${\left|f\left(\gamma \right)\right|}_{m}^{2}=\{\;\begin{array}{c} {\left(\frac{\Lambda }{2}\left(r_{1}+r_{2}\right)\right)}^{2}\;\;\;;\;\;m=0\\ {\left(\frac{\Lambda }{m\pi }\left(r_{1}-r_{2}\right)\right)}^{2};\;\mathrm{for}\;\mathrm{odd}\;m\;\\ \;\;\;\;\;0\;\;\;\;\;\;\;\;\;;\;\;\mathrm{for}\;\mathrm{even}\;m \end{array}\;.$$

The diffraction efficiency as defined by Equation (2) is then given as

(8) $$\eta_{\pm 1}=\frac{c^{2}}{2c^{2}+{\left(\pi /2\right)}^{2}}\;,$$

where *c* is the reflectivity contrast defined as

(9) $$c=\frac{r_{1}-r_{2}}{r_{1}+r_{2}}\;$$

In accordance with this model, it follows that*η*_−1_ = *η*_+1_ and consequently <*η*> = *η*_±1_. The resulting dependence of <*η*> on *c* as described by Equation (8) is shown as a solid line in Figure 7b. The value of <*η*> monotonically decreases with the decreasing contrast and becomes zero when *c* = 0, which happens when surface roughness over the entire surface becomes homogeneous; therefore, the grating pattern vanishes.

Equation (8) can be inverted to calculate *c* from the experimentally obtained values of <*η*>. The resulting dependence of *c*(*B*) for sample M2 is shown in the inset of Figure 7b. The contrast decreases with the decreasing value of *B*; however, it does not completely vanish at *B* = 0. We observed that a complete erasure of the grating pattern spontaneously happens in about 24 h after the removal of the magnet from the sample. Alternatively, the erasure can be strongly stimulated by a repetitive approach and distancing of the magnet to/from the sample. 

## 4. Discussion

Our results demonstrate that the shape memory effect of MAEs enables imprinting of temporary surface micropatterns, which exist as long as the magnetic field is present and are spontaneously erased after the field is removed. Subsequently, the field can be applied again and either the same or another pattern can be imprinted. The associated surface microstructure is hence reconfigurable. Our results also show that the readout of the imprinted micropattern can be conveniently realized by illuminating the surface with a coherent optical beam and recording its far-field optical diffraction image in the reflection grating configuration. This method enables analysis of the spatial as well as the temporal modifications of the surface pattern.

The observed optical diffraction phenomena and the obtained surface topography images reveal that the embossed surface pattern does not entirely resemble the shape of the stamp used for embossing. Instead of a structure of periodic surface channels, as depicted in Figure 1, we obtained a structure with periodically modulated surface roughness. This result is attributed to the fact that the thickness and the depth of the slabs in the stamp (15 μm) are comparable to the size of the CIP microparticles (average diameter 4.5 μm) present in the material. Consequently, the spatial resolution of the embossing process is limited by the particle size. Better quality of the embossed replicas is, hence, to be expected if MAEs with smaller particles are used. Another effect that reduces the quality of the replicas is the adhesion of the stamp to MAE, which affects especially the detachment process of the stamp after the embossing. For softer MAE samples, we often noticed that small parts of the MAE material were torn away from the surface and remained trapped in the channels of the stamp. We found that this problem can be reduced by a slight tilting of the stamp during the embossing process. Further improvements are possible by surface coating of the stamp with a layer that decreases the adhesion. 

The reported technical solutions for elastomeric reconfigurable optical diffraction gratings were primarily related to microfluidic applications [52,53,54]. They relied either on purely mechanical deformation of PDMS gratings [55,56] for pressure and force measurements or on filling of a PDMS diffraction grating with a fluid sample for measuring the optical properties of fluid [52]. These technologies did not find wide application. Alternatively, combinations of liquid crystals with soft polymers have been employed to demonstrate light-responsive and electro-activated diffraction gratings [57]. In this work, we propose an alternative method for reconfiguring flexible diffraction gratings by the use of a magnetic field. Regardless of the above-mentioned imperfections observed in our experiments, the embossed grating structures induced optical diffraction with easily detectable and clearly resolvable diffraction maxima. This suggests that MAEs can be used for the fabrication of magnetically controllable diffractive optical elements (DOEs). Although DOEs are one of the most important and consequently also most researched units for manipulation of optical signals, reports on magnetically regulable DOEs are relatively rare. The majority of investigations involving soft materials are focused on phase modulation-type gratings whose refractive index is tuned with the magnetic field [58,59,60,61]. In contrast, magnetically regulable gratings reported in this work belong to the amplitude modulation-type gratings, which are more difficult to be realized. Reconfigurable ridge-type surface gratings can be fabricated also from conventional shape memory polymers, utilizing thermally regulable reversible shape changes [62,63,64]. These gratings can be erased by melting of crystalline regions within the sample. Their diffractive properties are similar to diffractive properties of our MAE gratings. However, most of the investigated gratings in conventional shape memory polymers have grating pitch in the range of the wavelengths of visible light, as the main focus of the associated research is tuning of the structural color [65,66].

In addition to being regulable by magnetic fields, MAEs are also mechanically very deformable, so they offer an additional possibility to regulate diffractive optical properties with mechanical strain and vice-versa to regulate the strain via diffractive optical response. We hence believe that the investigated phenomena might have some interesting implications in soft matter photonics and other soft matter-based technologies. Furthermore, the embossing of a surface relief grating and a non-destructive observation of its dynamics in time-varying magnetic fields can be used as a novel experimental method for the investigation of the magnetic-field-induced plasticity on the micrometer scale. 

## 5. Conclusions

It is shown that the field-induced shape memory effect in MAEs also works on the micrometer scale. However, it is not the relief which is transferred to the MAE surface, but the modulation of the surface roughness takes place. The surface appears to be smoothed out where the flat ridges of the stamp are pressed against the MAE surface. Nevertheless, the resulting effect can be utilized for realization of reconfigurable diffractive optical elements, because the reflection coefficients from smoothed and rough stripes differ. It is also observed that MAE samples with the higher content of iron filling performed better than the samples with the lower concentration of particles. The origin of this advantage of higher filled MAE sample is not completely clear yet, it may be related to the jamming phenomenon. Further research is required in order to optimize the composition of MAE samples with respect to the enhancement of the diffraction efficiency and to find effective procedures for fast erasure of embossed gratings. 

## Data Availability

The data reported in this work are openly available in Mendeley at https://doi.org/10.17632/6v4dx92bfm.1 (accessed on 15 December 2021). Additional data are available on request from the corresponding author.
